# Toxic epidermal necrolysis in lenalidomide treated patient with HIV

**DOI:** 10.1186/1471-2334-14-S3-O28

**Published:** 2014-05-27

**Authors:** V Mahalakshmi, M Krishnakanth, S Adikrishnan, Gayathri R Adithya, S Murugan, S Anandan, R Sudha

**Affiliations:** 1 Department of Dermatology, Venereology and Leprosy, Sri Ramachandra Medical College, Porur, Chennai, India

## Introduction

Toxic Epidermal Necrolysis (TEN) is a rare and acute life threatening mucocutaneous reaction characterized by extensive necrosis and detachment of the epidermis, most commonly drug induced. Lenalidomide is a derivative of thalidomide used in treatment of multiple myeloma, myelodysplastic syndromes. We present a case of TEN in lenalidomide treated patient of multiple myeloma with HIV.

## Case Report

A 58 year old male, a k/c/o chronic kidney disease and multiple myeloma presented to the OPD with diffuse redness and peeling of the skin of 3 days duration associated with intense burning sensation and erosions in the mouth and genitalia. Patient was started on T. Lenalidomide 16 days ago for multiple myeloma. In view of an episode of herpes zoster in the past HIV testing was done which was found to be positive. O/E- multiple erosions were present over the scalp, face trunk and genitalia. There was approximately 60-80% of epidermal detachment and SCORTEN was 4. Nikolsky’s sign positive.

## Discussion

Lenalidomide is used as an alternative for thalidomide for its lesser incidence of side effects. HIV infected individuals are deficient in the enzyme glutathione peroxidase which is essential for drug metabolism. Only two cases of SJS, TEN to lenalidomide have been reported so far. The case is presented for its rarity and association with HIV.

